# Production of high-resolution forest-ecosite maps based on model predictions of soil moisture and nutrient regimes over a large forested area

**DOI:** 10.1038/s41598-017-11381-z

**Published:** 2017-09-08

**Authors:** Qi Yang, Fan-Rui Meng, Charles P.-A. Bourque, Zhengyong Zhao

**Affiliations:** 1 Guangxi Key Laboratory of Forest Ecology and Conservation, College of Forestry, Guangxi University, Nanning, 530004 China; 2 Faculty of Forestry and Environmental Management, University of New Brunswick, Fredericton, New Brunswick, E3B 5A3 Canada

## Abstract

Forest ecosite reflects the local site conditions that are meaningful to forest productivity as well as basic ecological functions. Field assessments of vegetation and soil types are often used to identify forest ecosites. However, the production of high-resolution ecosite maps for large areas from interpolating field data is difficult because of high spatial variation and associated costs and time requirements. Indices of soil moisture and nutrient regimes (i.e., SMR and SNR) introduced in this study reflect the combined effects of biogeochemical and topographic factors on forest growth. The objective of this research is to present a method for creating high-resolution forest ecosite maps based on computer-generated predictions of SMR and SNR for an area in Atlantic Canada covering about 4.3 × 10^6^ hectares (ha) of forestland. Field data from 1,507 forest ecosystem classification plots were used to assess the accuracy of the ecosite maps produced. Using model predictions of SMR and SNR alone, ecosite maps were 61 and 59% correct in identifying 10 Acadian- and Maritime-Boreal-region ecosite types, respectively. This method provides an operational framework for the production of high-resolution maps of forest ecosites over large areas without the need for data from expensive, supplementary field surveys.

## Introduction

Ecosites, as stand-level units in ecological land classification systems, describe a suite of site conditions that characterize forest productivity. They also provide an ecological basis for grouping vegetation and soil types^1^. High-resolution ecosite maps (≤10 m) are useful for forest stand-level planning purposes, such as growth and yield analysis, best management practices implementation, and forest ecosystem management^2–4^. These maps provide forest managers, conservationists, and governmental organizations the ability to develop silvicultural systems, forest management plans, and environment-protection protocol and policy.

The principal method in identifying ecosites is based on gaging soil and vegetation types identified from a number of easily observable field indicators^5–8^. However, field procedures can be subjective in view of the fact that ground vegetation changes seasonally over the short-term and with forest stand succession over the longer term^9–11^. Also, from a mapping point of view, generating ecosite maps from the interpolation of point assessments would require many field surveys be carried out to produce maps of acceptable detail (i.e., resolution + accuracy), given the inherent complexity of forest landscapes. In general, field data-collection procedures associated with ecosite mapping are time consuming and expensive to use, particularly in large areas spanning more than ten to hundreds of thousands of hectares (ha). In the past two decades, new methods have been developed, largely based on air-photo- and model-based interpretation, with an aim to increase map production rates and reduce costs^12–14^. For example, MacMillan *et al*.^14^ reported that by mapping ecosites with automated-predictive-mapping procedures, total mapping costs for a 3 × 10^6^ ha forest, processed at a 25-m resolution, were reduced from more than $3.50 ha^−1^ to less than $0.20 ha^−1^. Work productivity was also seen to increase from less than 150,000 ha to more than 2 × 10^6^ ha per person per year. These outcomes inspired further work and innovation in this area of study.

Ecosites are influenced by climatic, biophysical, geological, and topographical factors^15–18^. A two-dimensional edatopic grid with soil moisture and nutrient regime classes (i.e., SMR and SNR) as coordinates are often used in classifying ecosites^19, 20^. The SMR and SNR classes are more operationally meaningful than plant-based indicators^6, 11, 21^. Here, SMR represents the average annual soil moisture available for plant growth, assessed by integrating moisture supply with soil drainage and moisture-holding capacities^7, 22^. Many factors, such as slope, aspect, slope gradients, slope position, soil texture, stoniness, depth, drainage and climate all have a role in influencing SMR^23–27^. Soil nutrient regimes, representing the relative availability of nutrients for plant growth^7^, is itself also affected by many of the same factors affecting SMR, including topography, geology, organic carbon, and harvesting history^28–31^. Traditionally, field recognizable factors (e.g., soil texture, soil drainage, soil structure, seepage class, ground water, together with indicator vegetation species) have been used to evaluate SMR and SNR^22, 32^. However, creating high-resolution SMR/SNR maps with conventional interpolation methods, like in the creation of ecosite maps discussed earlier, also requires a great deal of field information.

Previous research documented that soil properties, especially soil drainage, soil texture, and soil organic carbon (SOC), are close related to SMRs and SNRs^4, 33^. Soil drainage classes relate to the frequency and duration soils are saturated or partially saturated and reflect the average soil moisture regime of a soil^34^. Soil drainage is associated with soil water retention and hydrologic characteristics, solute transport, nutrient-holding capacities, and plant growth^35, 36^. Soil texture is defined as the relative proportion of clay to sand to silt content. Soil texture directly affects the porosity of soils, which in turn determines the water-retention and flow characteristics of the soil. Soil texture, especially the clay component, has a role in the soil’s nutrient-holding capacity and its long-term fertility^37^. Soil organic carbon helps to improve the soil’s physical and chemical properties by increasing its water-holding capacity and stabilizing structure^38^ and by improving the soil’s nutrient-holding capacity^39^.

Relating key soil properties with topo-hydrologic variables is complex, aggravated to the extent field data is missing. Fortunately, recent studies have shown that high-resolution soil properties, such as soil drainage, texture, and SOC can be estimated reasonably well at the watershed scale using a series of models to build the complex relation between soil properties and related topo-hydrologic variables^40–43^. A two-stage approach was developed to extend these models to large forest regions with a few field samples from a smaller area^33, 44^. This raises the possibility of producing ecosite classification maps without the use of time-consuming field surveys.

The main objective of this study was to develop method to map forest ecosites based on predictions of SMR and SNR over a large area. Specific objectives were to: (1) model SMR based on model predictions of soil property; (2) develop forest ecosite maps based on model predictions of SMR and SNR developed in a prior study; and (3) test the accuracy of the ecosite maps.

## Materials and Methods

### Study Area

**Figure 1:**
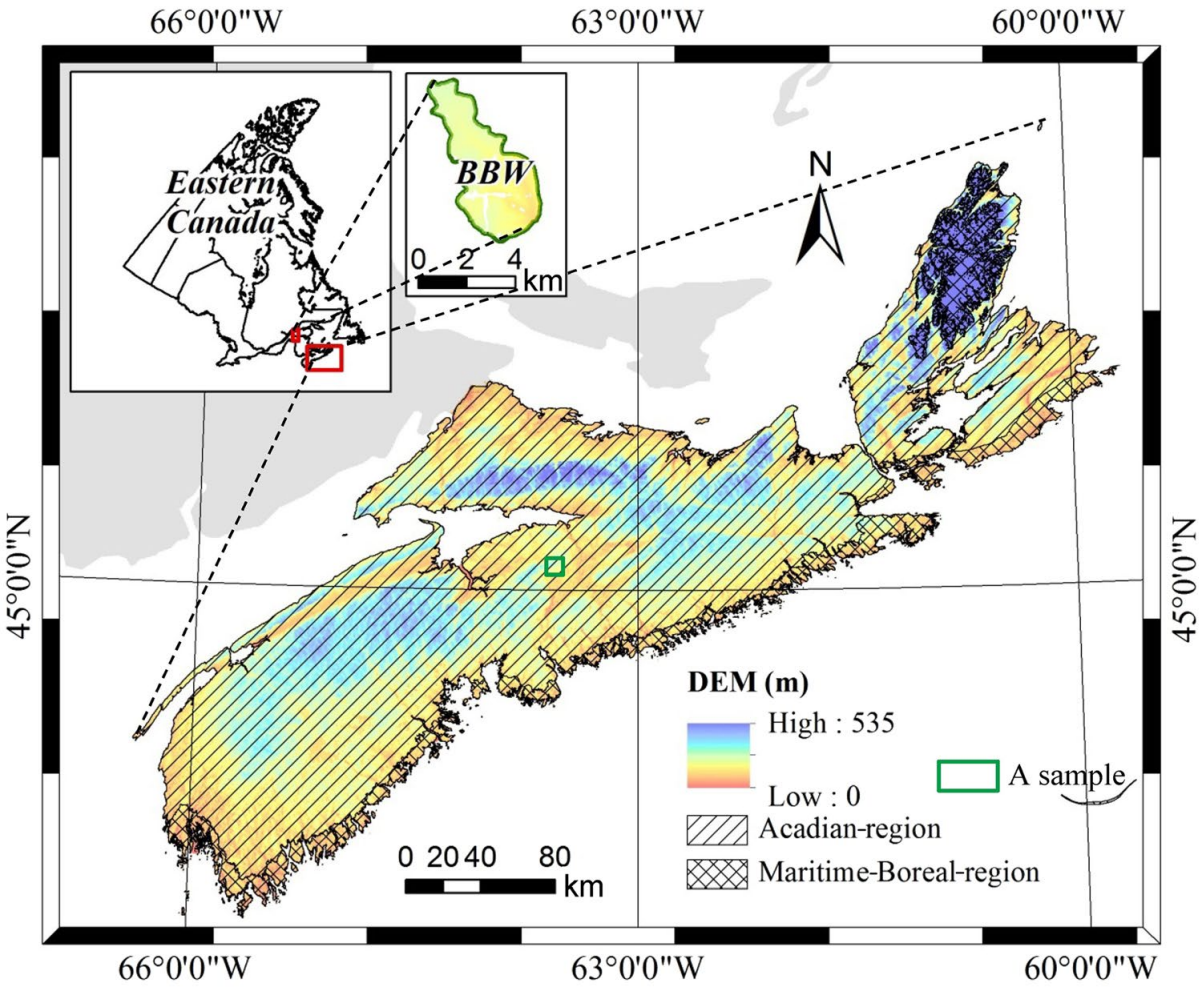
Digital elevation model (DEM) for Nova Scotia with Acadian-region and Maritime-Boreal region overlays and the Black Brook Watershed (BBW), New Brunswick, Canada. The map was generated by ArcGIS software (version 9.0; ESRI Inc.; http://www.esri.com/).

Nova Scotia is a maritime province located on the southeastern coast of Canada (Fig. 1; Lat. 43°25′-47°00N, Long. 59°40′-66°35′W). The total area of the province is approximately 5.5 × 10^6^ ha with 78% covered by forests. Nova Scotia lies in a mid-temperate zone, with mostly a continental climate with some influence from the Atlantic Ocean^45^. Different combinations of climate, topography, and parent material result in the development of a range of soil and associated vegetation types. The most common soils, derived from ablation and basal moraines, have nutrient and moisture conditions that vary with soil texture and topographic position. Organic soils occurring on wet sites usually have medium to poor soil nutrient content. Fluvial soils (e.g., deltas and floodplains) are generally nutrient rich with variable drainage properties, whereas glaciofluvial soils of variable parent material are usually nutrient poor^46–48^. The most common forest types in Nova Scotia have mixed-species composition that consists predominantly of conifers, i.e., spruce (*Picea* spp.), balsam fir (*Abies balsamea*), eastern hemlock (*Tsuga canadensis*), and pines (*Pinus* spp.). The major hardwood species in the region include maple (*Acer* spp.), birch (*Betula* spp.), aspen (*Populus* spp.), and American beech (*Fagus grandifolia*)^49^.

### Ecosites and Associated Sample Plots

Nova Scotia’s Ecological Land Classification system comprises of five descriptive orders, including ecozone, ecoregion, ecodistrict, ecosection, and ecosite. Ecozones and ecoregions capture macro-climatic influences. Ecodistricts and ecosections describe the variation in topographic pattern, landform, and soil parent material, whereas ecosites describe site conditions at the individual stand level^1, 7^. Large-scale classification maps, covering land information at the ecozone to ecosection scale, have been produced by the Nova Scotia Department of Natural Resources (NSDNR). This study provides an initial attempt to describe the same land base at the ecosite scale.

Field data for map evaluation were obtained from 1,507 fixed-area forest ecosystem classification (FEC) plots established by NSDNR between 2000 and 2010. These plots were strategically placed to cover the full range of SMR and SNR expected within the province in order to produce a robust FEC system^7^. At each plot, vegetation (total 88 vegetation types, within 14 forest groups) and soil type (19 soil types with six phases), and tree growth were routinely assessed^48, 49^. Assigning SMR and SNR to field plots was based on a FEC-assessment of observable parameters (Table 1)^4^. Classifying SNR *in situ* was rated according to five classes, namely very poor, poor, medium, rich, and very rich. Soil moisture regimes were classed according to very dry, dry, fresh, fresh/moist, moist, moist/wet, and wet.

**Table 1:** Soil moisture and nutrient regimes as a function of site and soil conditions (modified after Table 4 and Fig. 1 in Keys *et al*.^4^).

	Classes	General site and soil features
SMR	Very dry	Rapidly drained, coarse-textured and/or shallow soils not influenced by ground water or seepage; soil retains moisture for negligible duration following precipitation
	Dry	Deeper, well drained, coarse-textured soils not influenced by ground water or seepage; or shallow soils not rapidly drained; soil retains moisture for a brief duration following precipitation
	Fresh	Deeper, well drained, medium to fine textured soils; moderately well drained coarse textured soils; or well drained coarse textured soils; soil retains moisture for moderately short periods following precipitation
	Fresh/Moist	Deeper, moderately well drained, medium to fine textured sol often with some evidence of anaerobic (reducing) conditions in lower BC and C horizons; soil retains moisture for substantial periods following precipitation
	Moist	Soils with imperfect drainage, but with surface soil still well aerated during most of the growing season. Evidence of anaerobic (reducing) conditions in upper B horizons; soil is wet for a substantial part of the growing season
	Moist/Wet	Poorly drained soils with water levels at or near the surface for most of the year, but with well aerated surface conditions during dry periods
	Wet	Very poorly drained soil with water levels at or above the surface for most of the year (often associated with wetland organic soils)
SNR	Very poor	Shallow, coarse textured soil with mor humus form, Ae horizon, and stagnant ground water but not influenced by seepage
	Poor	Shallow or moderate, coarse or medium textured soil with mor humus form, Ae/Ahe horizon, stagnant ground water and minor potential for seepage influence
	Medium	Shallow or moderate to deeper, coarse or medium to fine textured soil with Ae/Ahe/Ah/Ap horizon and influenced by ground water or seepage
	Rich	Moderate to deeper, medium to fine textured soil with modor/mull humus form, Ahe/Ah/Ap horizon, moving ground water and major potential for seepage influence
	Very rich	Moderate to deeper, fine textured soil with mull humus form, Ah/Ap horizon, moving ground water and major potential for seepage influence

In the field, ecosite is determined by assessing stand-level vegetation and soil types (VT and ST) using an ecosite-matrix approach developed by NSDNR^4^. Assignment to combinations of VT/ST was based on expert opinion and analysis of tree growth data from FEC plots^4^. Based on tree growth patterns, NSDNR has sub-divided the province into two main ecological regions, i.e., the Acadian- and Maritime-Boreal-region (Fig. 1), displaying fundamentally different climatic and productivity regimes^4^. In general, sites in the Acadian-region tend to be associated with higher land capability values (LC; in m^3^ ha^−1^ yr^−1^) compared to sites in the Maritime-Boreal-region with similar SMR, SNR and forest type. For instance, LC decreases from 2.8 (Acadian-region) to 2.0 (Maritime-Boreal-region), 1.9 to 1.0, and 2.5 to 1.3 m^3^ ha^−1^ yr^−1^ for black spruce (*Picea mariana*), balsam fir (*Abies balsamea*), and white spruce (*Picea glauca*) associated sites, respectively.

### Simplification of Ecosite Classification

**Figure 2:**
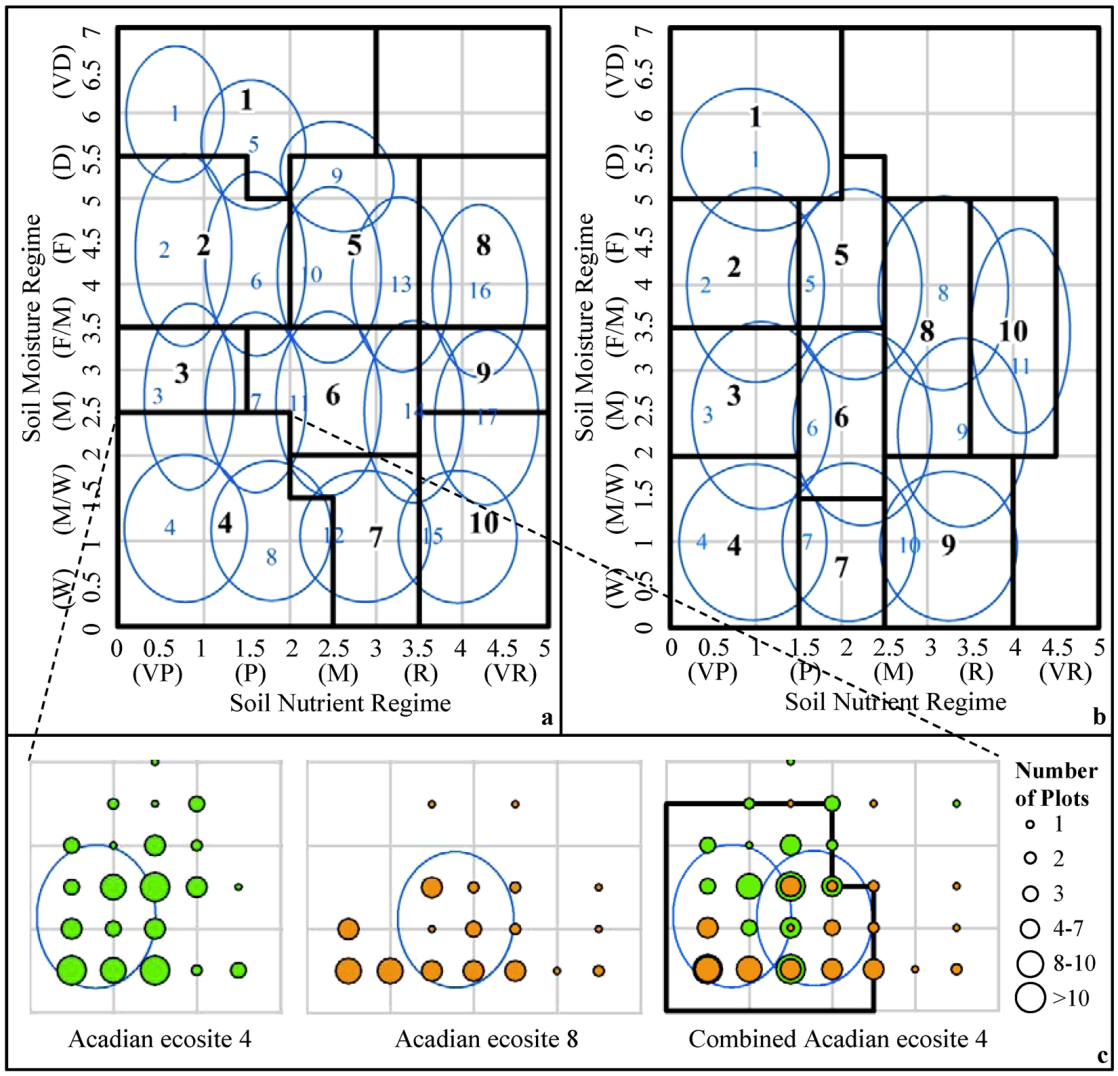
Comparison of new edatopic grids (based on the black-outlined rectangles) showing relative moisture and nutrient regimes for 10 Acadian- (**a**) 10 Maritime-Boreal-region ecosites (**b**) of Nova Scotia and the original edatopic grids (based on the system of blue ellipses) being centred on 17 Acadian- and 11 Maritime-Boreal-region ecosites identified in the field^4^, and the combining and transforming procedure of Acadian ecosite 4 and 8 with FEC plots (**c**).

The ecosites in the Maritime-Boreal-region are found mainly along the Atlantic coast and in the Cape Breton Highlands (accounting for about 12% of all provincial forestland) with the remaining sites being more closely associated with the Acadian-region. There is a total of 17 ecosites in the Acadian-region and 11 ecosites in the Maritime-Boreal-region^4^. The 28 ecosites are classified according to their positions in an edatopic grid (two-dimensional grid), with relative moisture (SMR) defining the vertical axis and nutrient availability (SNR) as the horizontal axis (Fig. 2, one for each ecological region). Different ecosite classifications are identified as blue-outlined ellipses based field experiences^4^. Clearly, some overlap exists between ecosite types due to variations in vegetation types, potentially as a result of stand succession and intervention. These overlaps serve the purpose for field assessment that have access to information about understory indicator plant species. However, this overlap could cause confusion when characterizing ecosites using digital map without assess of information of ground vegetation. For example, Acadian-region ecosite 4 (dominated by black spruce, jack pine, and red pine; blue numbers, lower left-hand-side of Fig. 2a) can be clearly distinguished from ecosite 8 (red spruce, balsam fir, tamarack, hemlock, and red maple) when using vegetation type as primary indicator of ecosite. However, this distinction becomes less obvious when ecosite is based on SMR and SNR. Based on analysis of FEC plots, 36 of 101 FEC plots that fell inside the ellipse for Acadian-region ecosite 8 (Fig. 2c) should have been identified as ecosite 4, and 23 of 63 FEC plots that fell inside the ellipse for ecosite 4 should have been identified as ecosite 8.

In order to map ecosites as correctly as possible with SMR and SNR as primary indicators, mixed ecosites were combined and transformed giving the black-outlined system of rectangles in Fig. 2a (Acadian-region ecosites) and 2b (Maritime-Boreal-region ecosites) based on the field data from FEC plots. The combining procedure aimed to replace mixed ecosites with simplified ecosites so that most of the plots could be properly identified only according to their SMR and SNR classes in the original edatopic gird, with three conditions (Table 2): neighbouring ecosites in original edatopic gird only were distinguished by field-assessed soil type and vegetation type (Condition 1), by field-assessed soil type (with similar vegetation type) (Condition 2), by field-assessed vegetation type (with similar soil type) (Condition 3), but not done by field-assessed SMR and SNR classes, so all of neighbouring ecosites could be combined as one ecosite. For example, after combined ecosite 4 and 8, the new 36 + 23 plots fell inside the combined ellipse (Fig. 2c). This transforming procedure aimed to replace the systems of overlapping ellipses with non-overlapping rectangles so that most of the plots could be properly identified according to their positions in the new edatopic grid. Based on the analysis of FEC plots, each overlapped ellipses was transformed into rectangles along the horizontal (SMR) and vertical (SNR) axis in edatopic grid with including as many plots as possible, but not affecting neighbouring ecosites as little as possible. For example, after transformed the combined ellipse 4 into rectangle, the new 16 plots fell inside the rectangle ecosite 4 (Fig. 2c).

**Table 2:** Comparison of ecosites as defined in Keys *et al*.^4^ and combined ecosites with related conditions and associated combinations.

	Ecosites according to Keys *et al*.^4^		Combined ecosites	
	Ecosites	Forest type	Ecosites	Conditions for combinationz
Acadian-region	1	Jack pine-Black spruce	1	Condition 1
	5	White pine-Oak		
	9	Red maple-Spruce		
	2	Black spruce-Pine	2	Condition 2
	6	Black spruce-White pine		
	3	Black spruce-Pine	3	
	4	Black spruce-Tamarack	4	Condition 3
	8	Spruce-Fir-Red maple		
	10	Red spruce-Hemlock	5	Condition 2
	13	Sugar maple-Beech		
	7	Black spruce-White pine	6	Condition 3
	11	Red spruce-Yellow birch		
	14	Sugar maple-Yellow birch		
	12	Red maple-White ash-Fir	7	
	16	Sugar maple-White ash	8	
	17	Sugar maple-White ash	9	
	15	White ash-Red maple	10	
Maritime-Boreal-region	1	Black spruce-Jack pine	1	
	2	Black spruce	2	
	3	Black spruce	3	
	4	Black spruce	4	
	5	Fir-Spruce	5	
	6	Fir-Spruce	6	
	7	Red maple-Fir	7	
	8	Birch-Fir	8	Condition 2
	9	Birch-Fir		
	10	Red maple	9	
	11	Red maple-Birch	10	

^z^Condition 1. neighbouring ecosites in original edatopic gird only were distinguished by field-assessed soil type and vegetation type but not done by field-assessed SMR and SNR classes, so all ecosites could be combined as one ecosite; Condition 2. neighbouring ecosites in original edatopic gird only were distinguished by field-assessed soil type (with similar vegetation type) but not done by field-assessed SMR and SNR classes, so all ecosites could be combined as one ecosite; Condition 3. neighbouring ecosites in original edatopic gird only were distinguished by field-assessed vegetation type (with similar soil type) but not done by field-assessed SMR and SNR classes, so all ecosites could be combined as one ecosite.

### Maps of Model Predictions of Soil Moisture and Nutrient Regime

**Figure 3:**
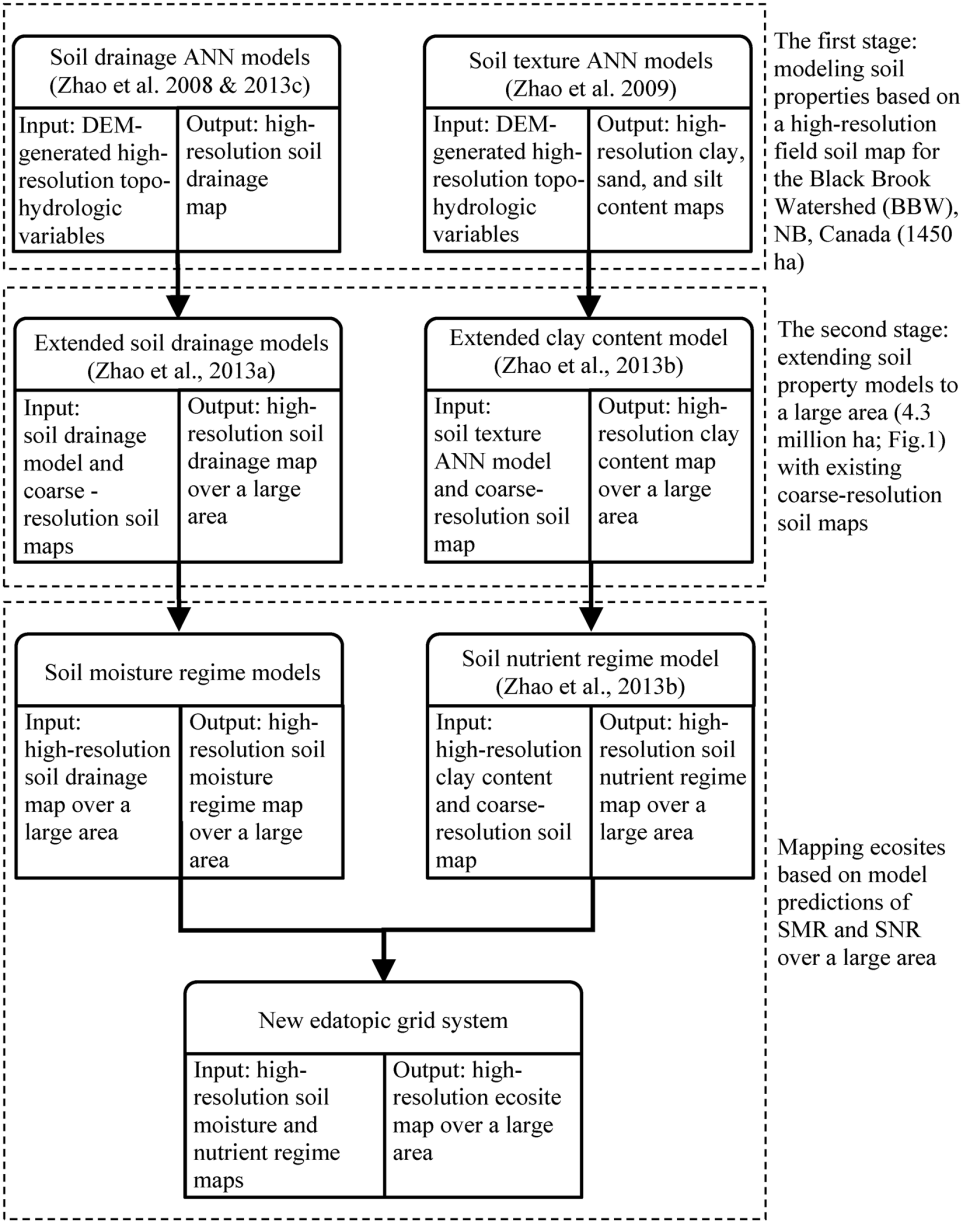
Schematic diagram showing model-predicted SMR and SNR for mapping high-resolution ecosites over a large forested area.

Figure 3 presents a schematic of information flow required in mapping high-resolution SMR and SNR over a large area. A two-stage approach was developed to produce key soil properties (soil drainage + texture) maps for the same area. During this initial stage, existing soil property models developed for a small experimental watershed in northwestern New Brunswick (Fig. 1) were adopted^41, 42, 50^ for local conditions. The small watershed (denoted as BBW in Fig. 1) was ideal in developing these models because of the availability of ample field information. During the second stage, after dividing the large study area into sub-areas based on existing coarse soil maps, corresponding linear-transformation models were subsequently developed to adjust soil properties produced by a base model (existing soil property model) according to local field samples. These models were then used to produce high-resolution soil property maps for the study area^41, 42^ as basis for the development of SMR and SNR maps.

A map of SMR was produced from model predictions of soil drainage^44^ with two steps. In the first step, linear regression equations between SMR and soil drainage values were developed from 1,507 FEC-plot assessments of SMR and soil drainage classes as a summary of existing dataset, after the ordinal-scaled SMR and soil drainage classes were converted into continuous variables: a numerical value for the seven-class soil drainage system (i.e., 0.5-very poor, 1.5-poor, 2.5-imperfect, 3.5-moderately well, 4.5-well, 5.5-rapidly, and 6.5-very rapidly drained), for the seven-class SMR (i.e., 0.5-wet, 1.5-moist/wet, 2.5-moist, 3.5-fresh/moist, 4.5-fresh, 5.5-dry, and 6.5-very dry) and the middle values being given to the half classes (i.e., 1.0-very poor/poor) that often appear on the plots where it’s hard to clearly identify the optimal classes between neighbouring two classes. In the second step, linear regression equations were applied to produce a SMR map from an independent model prediction of soil drainage that were derived from a digital elevation model (DEM) with a 10-m resolution using an developed artificial neural network (ANN) model^44^. The soil drainage map used here had an overall accuracy of 36% of model predictions being the same as the field assessments, and 84%, withi ±1 drainage class^44^. It is noteworthy that model prediction of soil drainage was continuous value, which explained that why linear regression equations between two ordinal-scaled SMR and soil drainage were developed. Thus, the value of produced SMR using linear regression equations also was continuous value. However, when assessing an accuracy of the produced SMR map using 1,507 FEC-plot assessments, the values of predicted SMR should be reconverted into ordinal-scaled SMR classes (i.e., 0.1-1.0-wet, 1.1-2.0-moist/wet and so on; when considering half classes, 0.0-0.25-wet, 0.26-0.75-wet/moist/wet, 0.7601.25-moist/wet and so on). The SNR map, also being continuous value, was generated in a prior study from model predictions of clay content that were derived from the same DEM like soil drainage map^33^. The original irregular elevation points were compiled from Satellite Pour l’Observation de la Terre (SPOT 5) images taken at 1 to 27-m intervals (courtesy of Nova Scotia Department of Nature Resources). The production of the DEM was based on an interpolation of elevation data with the inverse distance weighted method (Fig. 3)^51^. To provide a comparable accuracy with SMR map and previous research, 1,507 FEC-plot assessments also be used to re-assess the independent model prediction of SNR map after the values of predicted SNR were reconverted into ordinal-scaled SNR with five classes.

### Forest Ecosite Mapping

Two-step process was used to determine the ecosite classes. First, the above methods were used to determine the values of SMR and SNR, both continuous values, at the sample plot. Information pertaining to SMR and SNR was then used to project the sample plot onto the transformed edatopic grid and assign an ecosite designation that best matched its position on the grid^52^. For example, if values of SMR and SNR for a particular plot of the Acadian-region were confirmed to be 3.6 and 0.5, the plot would be assigned to Acadian-region ecosite 2, in accordance with the information in Fig. 2a. It is noteworthy that if value of SMR for another particular plot was 3.4 and value of SNR did not change, the plot would be assigned to Acadian-region ecosite 3. It said that continuous values of SMR and SNR make sense to this study, which would assign an ecosite designation more accurate than being done by SMR and SNR classes (ordinal-scaled values). In the example, if using SMR and SNR classes to assign ecosite designation on the edatopic grid, the two plots would be assigned to the same ecosite because ordinal-scaled fresh/moist classes of SMR corresponds a range from 3.0 to 4.0 for continuous values of SMR (or a range from 3.25 to 3.75 for continuous values of SMR if considering half classes). The same procedure was used to give ecosite designations for every 10 m × 10 m grid cell (equivalent to the size of a single FEC plot) when creating the ecosite map for Nova Scotia.

### Accuracy Assessment

Field assessments of ecosites at the individual FEC-plot level based on field assessments of vegetation and soil types, total 1,507 plots, were then used to assess the accuracy of gridded, ecosite classifications resulting from the broad-scale mapping for the study area, which was labelled as model-prediction accuracy. As a contrast, the FEC plots were projected onto the transformed edatopic gird (black-outlined rectangles in Fig. 2a,b) to assign a new ecosite designation based on their field-assessed SMR and SNR classes. The accuracy of reassigned ecosites against field-assessed ecosites was labelled as rectangle-based accuracy. When the FEC plots were projected onto the original edatopic gird (blue-outlined ellipses in Fig. 2a,b by Key *et al*. 2010) to assign a new ecosite designation based on their field-assessed SMR and SNR classes, the accuracy of reassigned ecosites against field-assessed ecosites was labelled as ellipse-based accuracy.

## Results and Discussion

### New edatopic grids

Table 3 gives the level of ellipse-based accuracy, compared with rectangle-based accuracy. For the 17 Acadian-region ecosites, a total of 59% FEC plots correctly fell within the specified ellipses (blue-outlined ellipses in Fig. 2a,b). Only 4 ecosites were correctly identified for more than 65% of the time. The 11 Maritime-Boreal-region ecosites were correctly identified for 65% of FEC plots, with accuracies ranging from 0 to 100%. Based on the new edatopic grids (black-outlined rectangles in Fig. 2a,b), total accuracy improved to 84% (with an increase of 25%, on average) for the Acadian-region ecosites and to 80% (with an increase of 18%, on average) for the Maritime-Boreal-region ecosites. Combining ecosites generally improved the representation of ecosites across Nova Scotia.

**Table 3:** Comparison of ellipse-based accuracy based on original edatopic gird defined by Keys *et al*.^4^ with rectangle-based and model-prediction accuracies based on transformed edatopic grid.

	Original edatopic gird from Keys^4^				Transformed edatopic gird for this study					
	Ecosite type	Total plots	Ellipse-based		Ecosite type	Total plots	Rectangle-based		Model-prediction	
			Within plotsz	Accuracy (%)^y^			Within plots^x^	Accuracy (%)	Within plots^w^	Accuracy (%)
Acadian-region	1	28	16	57	1	90	65	72	21	23
	5	33	18	55						
	9	29	2	7						
	2	43	15	35	2	164	136	83	96	59
	6	121	89	74						
	3	13	9	69	3	13	9	69	5	39
	4	101	48	48	4	164	149	91	71	43
	8	63	26	41						
	10	289	208	72	5	534	467	87	192	71
	13	245	146	60						
	7	70	43	61	6	271	220	81	23	30
	11	108	67	62						
	14	93	58	62						
	12	76	49	65	7	76	60	79	22	46
	16	48	16	33	8	48	40	83	404	76
	17	13	4	31	9	13	11	85	6	46
	15	11	6	55	10	11	11	100	1	9
	Total	1384	820	59	Total	1384	1168	84	841	61
Maritime-Boreal-region	1	4	4	100	1	4	4	100	4	100
	2	11	5	46	2	11	6	55	2	18
	3	11	6	55	3	11	9	82	8	73
	4	16	12	75	4	16	14	88	9	56
	5	31	27	87	5	31	31	100	21	68
	6	16	10	63	6	16	15	94	15	94
	7	7	4	57	7	7	4	57	4	57
	8	16	6	38	8	21	12	57	8	38
	9	5	0	0						
	10	2	1	50	9	2	1	50	1	50
	11	4	1	25	10	4	2	50	1	25
	Total	123	76	62	Total	123	98	80	73	59

^z^Number of plots that full fall within specific ecosites (blue-outlined ellipses) in original edatopic gird (Fig. 4) base on their field-assessed SMR and SNR classes; ^y^calculated by dividing the number of plots that fall within specific ecosites by the total number of plots; ^x^number of plots that full fall within specific ecosites (black-outlined rectangles) in transformed edatopic gird base on their field-assessed SMR and SNR classes. ^w^Number of plots that full fall within specific ecosites (black-outlined rectangles) in transformed edatopic gird base on their model prediction of SMR and SNR values.

### Model-predicted Soil Moisture Regime and Soil Nutrient Regime maps

**Figure 4:**
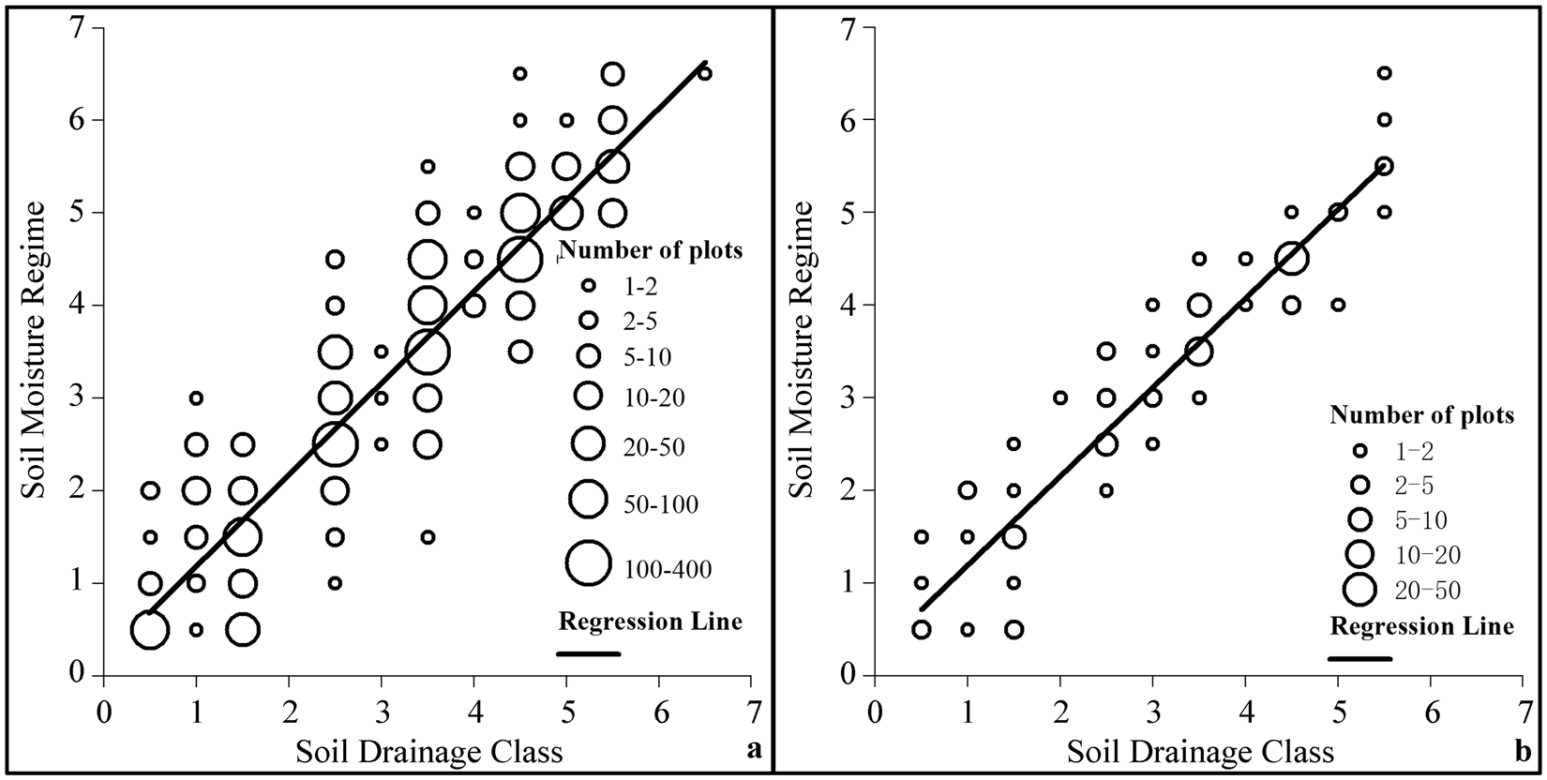
Relationship between soil drainage classes and soil moisture regimes based on 1384 forest ecosystem classification plots from Acadian-region (**a**) and 123 forest ecosystem classification plots from Maritime-Boreal-region (**b**) and associated regression lines.

Based on field-assessments of SMR and soil drainage classes from 1,384 FEC plots from the Acadian-region, parameters in Eq. 1 were estimated using linear regression analysis (Fig. 4a; coefficient of determination (*r* ^2^) = 0.88). Parameters in Eq. 2 were estimated based on 123 FEC plots from the Maritime-Boreal-region (Fig. 4b; *r* ^2^ = 0.89).

1 $$SMR=0.1997+0.9869SD$$

2 $$SMR=0.2683+0.9500SD$$

where *SD* represents a numerical value for the seven-class soil drainage system (0.5-very poor, 1.5-poor, 2.5-imperfect, 3.5-moderately well, 4.5-well, 5.5-rapidly, and 6.5-very rapidly drained) and *SMR*, predicted value of the seven-class SMR (i.e., 0.5-wet, 1.5-moist/wet, 2.5-moist, 3.5-fresh/moist, 4.5-fresh, 5.5-dry, and 6.5-very dry).

An error matrix of accuracy was constructed using FEC-plot assessments and model predictions of SMR (Table 4). Forty-seven to 58% of model predictions of SMR were the same as field assessments with 83 to 91% within ±1 class. Accuracy of the SMR map produced was a little lower than the reported 55% overall agreement, and 94% agreement, within ±1 class, based on plant indicators of SMR^28^. For the Acadian-region, most of the plots classified in the field as being very dry and dry were projected as being dry and fresh, respectively. Most of the plots classified *in situ* as being wet and moist/wet were projected as being moist. Results show that predictions of SMR were mostly clumped in the middle of the SMR classes, resulting in more plots being projected as moist (722 vs 614). A slightly better result was found for the Maritime-Boreal-region, but the clumping noted earlier still persisted. These results are not totally unexpected given that the predictions of SMR would have inherited input errors associated with the soil drainage maps. In assessing their soil drainage model, Zhao *et al*.^41^ reported that areas with extreme drainage conditions (e.g., very rapidly and very poorly drained areas) were poorly replicated, with <20% accuracy, as these drainage conditions cannot be represented by topographical indicators alone. The map is the first high-resolution map of SMR for the province of Nova Scotia. Furthermore, Fig. 2 indicates that most ecosites exist in more than one SMR classes. For example, SMR for Acadian-region ecosites 4 range from being wet to moist. Thus, 83-91% of model-predicted SMRs being within ±1 class of field assessment should be suitable for mapping ecosites in this study.

**Table 4:** Error matrices of soil moisture regime.

Region	Field data	Model predictions^z^								Producer’s accuracy^y^ (%)	
		W	MW	M	FM	F	D	VD	Total	Correct	within ±1 class
Acadian-region	W	**24**	17	33	23	12	0	2	111	22	37
	MW	3	**16**	40	39	17	1	0	116	14	50
	M	0	3	**69**	62	48	0	1	183	38	73
	FM	0	2	44	**76**	143	1	0	266	29	99
	F	1	2	39	119	**446**	7	0	614	73	93
	D	0	0	3	10	52	**18**	1	84	21	85
	VD	0	0	1	0	4	5	**0**	10	0	40
	Total	28	40	229	329	722	32	4	1384	47^x^	83^w^
Maritime-Boreal-region	W	**3**	1	4	0	0	0	0	8	38	50
	MW	0	**8**	2	3	0	0	0	13	62	77
	M	0	0	**11**	7	1	0	0	19	58	95
	FM	0	0	2	**19**	3	1	0	25	76	96
	F	0	0	2	18	**28**	3	0	51	55	96
	D	0	0	0	0	4	**2**	0	6	33	100
	VD	0	0	0	0	0	1	**0**	1	0	100
	Total	3	9	21	47	36	7	0	123	58 ^x^	91 ^w^

^z^soil moisture regime classes/SMR values: wet (W)/0.0–1.0, moist/wet (MW)/1.1–2.0, moist (M)/2.1–3.0, fresh/moist (FM)/3.1–4.0, fresh (F)/4.1–5.0, dry (D)/5.1–6.0, and very dry (VD)/6.1–7.0; ^y^Producer’s accuracy is a metric used to evaluate individual classes; an assessment of “correct” was calculated by dividing the total number of correctly predicted plots (bold cells) by the total number of plots in the same row; the value of within ±1 class was calculated by dividing the total number of predicted plots within ±1 class (bold and underlined cells) by the total number of plots in the same row; ^x^calculated by dividing the total correctly predicted plots (bold cells) by the total number of plots; ^w^calculated by dividing the total number of plots within ±1 class (bold and underlined cells) by the total number of plots involved.

With respect to SNR, 57–68% of model predictions were identical to field assessments with 98–100%, within +1 class (Table 5). This prediction accuracy was similar to that obtained by Wang^28^, who reported a 59% overall agreement and 97% agreement, within ±1 class, when using plant-based indicators. In the Acadian-region, most plots assessed *in situ* as being very poor, poor, and very rich plots were projected as poor, medium, and rich, respectively, indicating the challenge of modelling SNR in these special areas with coarse-resolution soil information and predicted clay content alone. In spite of this, 98–100% agreement, within ± 1 class of field assessments, should provide a reasonable basis for mapping ecosites because, like SMR, most ecosites are distributed across a range of SNR.

**Table 5:** Error matrices of soil nutrient regime.

Region	Field data	Model predictions^z^						Producer’s accuracy^y^ (%)	
		VP	P	M	R	VR	Total	Correct	within ±1 class
Acadian-region region	VP	**6**	36	16	0	0	58	10	72
	P	4	**152**	185	9	0	350	43	97
	M	2	57	**513**	187	0	759	68	100
	R	0	3	61	**119**	18	201	59	99
	VR	0	0	4	9	**3**	16	19	75
	Total	12	248	779	324	21	1384	57^x^	98^w^
Maritime-Boreal-region	VP	**4**	9	0	0	0	13	31	100
	P	2	**32**	14	0	0	48	67	100
	M	0	10	**46**	1	0	57	80	100
	R	0	0	3	**2**	0	5	33	100
	VR	0	0	0	0	**0**	0	—	—
	Total	6	51	63	3	0	123	68^x^	100^w^

^z^soil nutrient regime classes/SNR values: very poor(VP)/0.0–1.0, poor(P)/1.1–2.0, medium(M)/2.1–3.0, rich(R)/3.1–4.0, and very rich(VR)/4.1–5.0. ^y^Producer’s accuracy is a metric used to evaluate individual classes; an assessment of “correct” was calculated by dividing the total number of correctly predicted plots (bold cells) by the total number of plots in the same row; the value of within ±1 class was calculated by dividing the total number of correctly predicted plots within ±1 class (bold and underlined cells) by the total number of plots in the same row; ^x^calculated by dividing the total correctly predicted plots (bold cells cells) by the total number of plots; ^w^calculated by dividing the total number of plots within ±1 class (bold and underlined cells) by the total number of plots involved.

### Mapped Forest Ecosites

**Figure 5:**
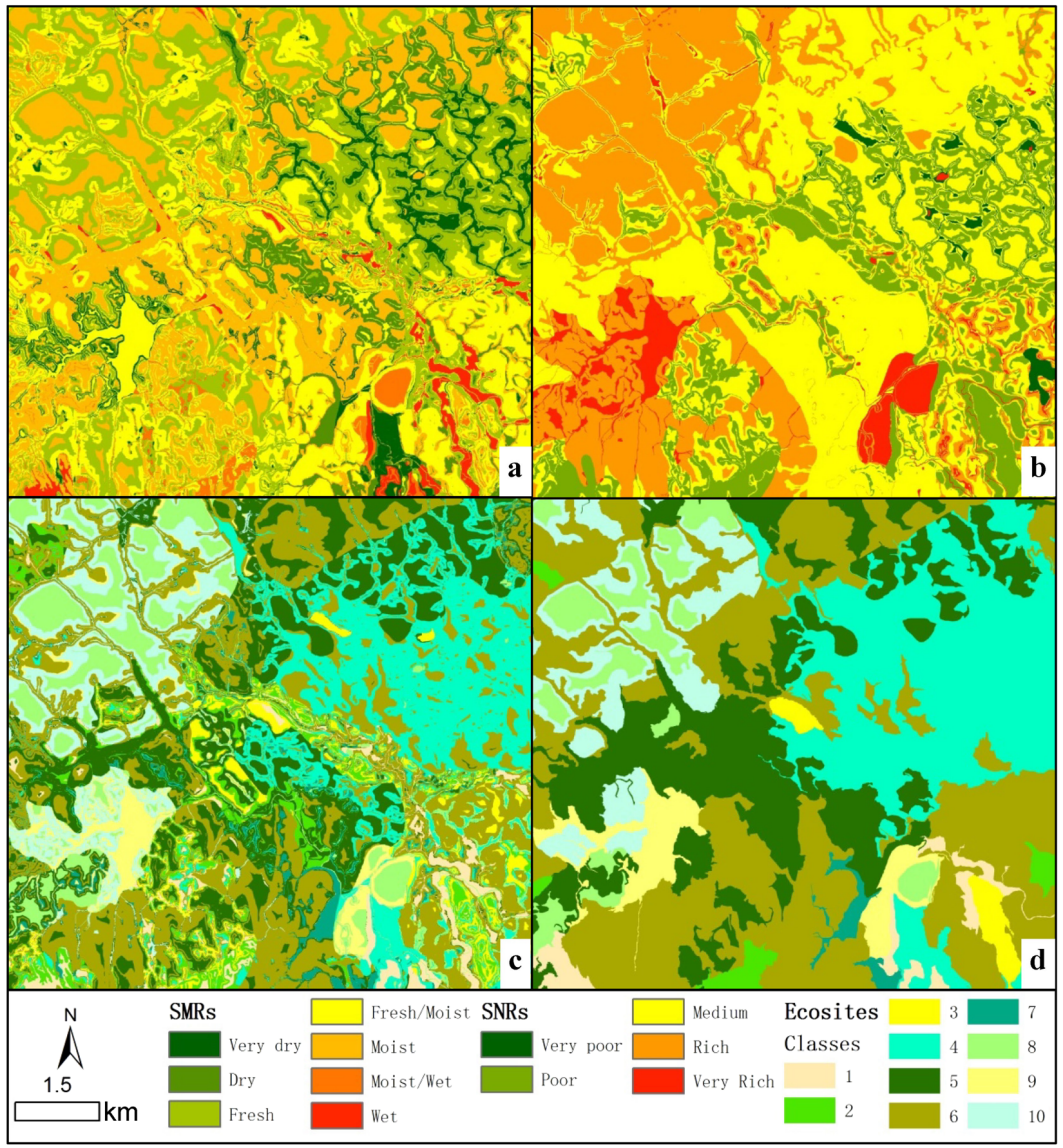
A sample of model predictions of SMR (**a**) SNR (**b**) ecosites (**c**) and ecosites eliminated the polygons with less than 25 ha area (**d**).

Accuracy of Acadian- and Maritime-Boreal-region ecosite maps derived from model predictions of SMR and SNR are shown in Table 3. For 10 Acadian-region ecosites, model-prediction accuracy had a total of 61% correctness, ranging from 9 to 76%. Ecosites located at the centre of the edatopic grid (e.g., ecosites 5 and 6) were better represented than those offset from the centre (e.g., ecosites 1, 3, 7, and 10). The 10 Maritime-Boreal-region ecosites were accurately replicated for 59% of the plots, ranging from 18 to 100%. Except ecosite 1, the ecosites in the middle of the edatopic grid (e.g., ecosite 6) were better represented than most along the edges (e.g., ecosites 2 and 10). A sample of mapped forest ecosites associated with model predictions of SMR and SNR was showed at Fig. 5.

**Figure 6:**
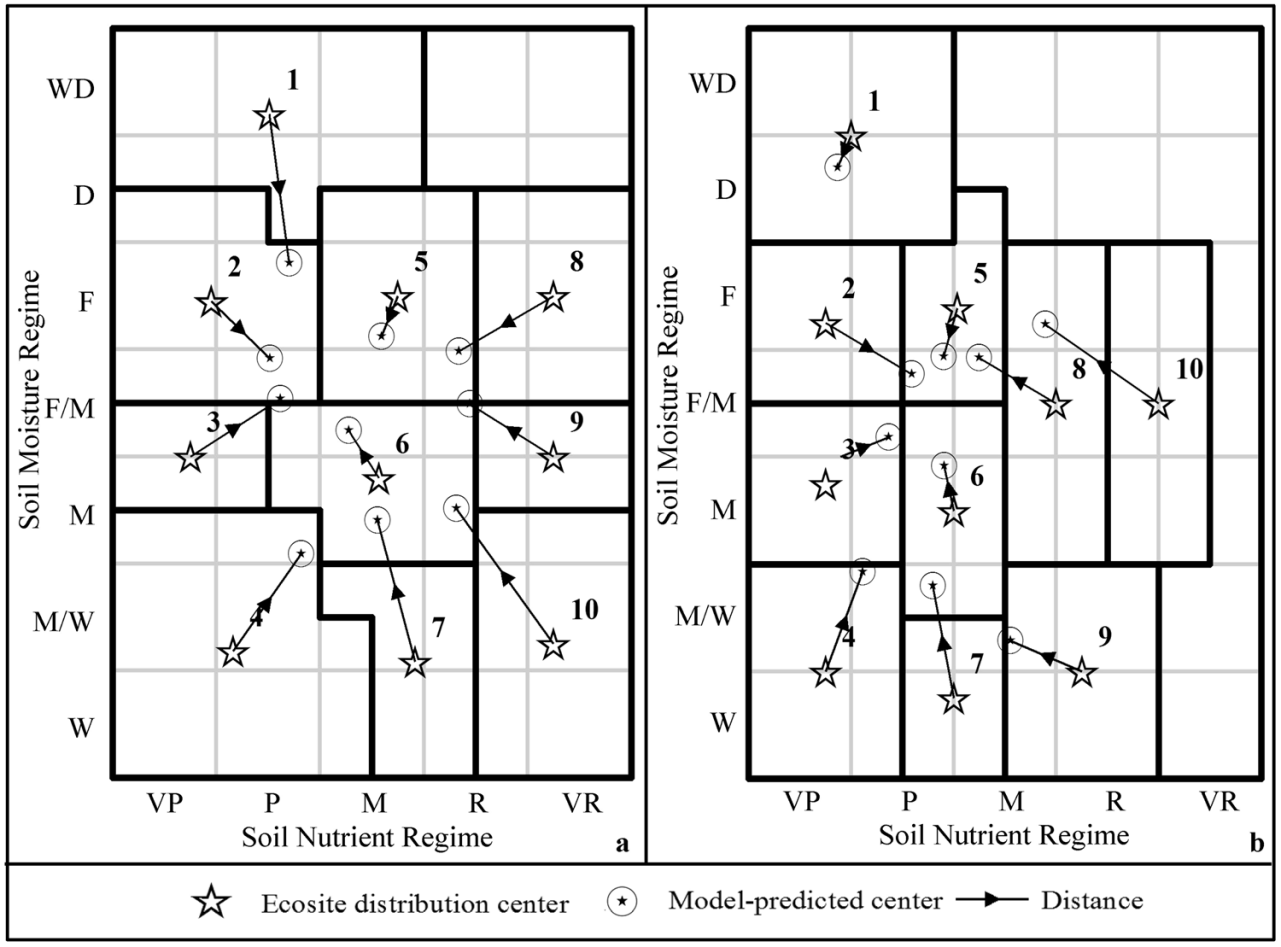
Comparison of geometrical centers of the plotted ecosite distributions (ecosite distribution centers) and average model-predicted SMR and SNR values of field-assessed ecosites (model-predicted centers) and associated the distances between the ecosite distribution center and the model-predicted center within the edatopic gird for 10 Acadian-region ecosites (**a**) and 10 Maritime-Boreal-region ecosite (**b**).

Based on analysis of plot data, the main reason for the variation in accuracies is attributable to problems in model predictions of SMR and SNR, especially in areas of extreme conditions. Figure 6 compares, plotted on the edatopic grid, the geometrical centers of plotted ecosite distributions from Fig. 2 (ecosite distribution centers) and the average model predictions of SMR and SNR of field-assessed ecosites (model-predicted centers). We expected that the ecosite distribution centers should overlap model-predicted centers if field-assessed ecosites were similar to those determined from model-predicted SMR and SNR. However, the distances between plotted ecosite distribution centers and model-predicted centers (Fig. 6a,b) showed that differences existed between the two ecosite distributions. Furthermore, model-predicted centers within edatopic grids generally converged at a central square for both Acadian-region and Maritime-Boreal-region ecosites (Fig. 6a,b). These observations indicated that model-predicted SMR and SNR have difficult to capture the extreme moisture/nutrient conditions (e.g. Wet or Very Dry, Very Poor or Very Rich) sampled in the field, and model-predicted SMR/SNR were clumped into the middle of SMR/SNR classes, which is in keeping with the analysis of accuracy assessment error discussed above (Tables 4 and 5).

Although total 59–61% accuracy of model-predicted ecosite maps with 10-m resolution were little lower than the 66–70% accuracy reported by MacMillan *et al*.^14^ for predicting ecosite maps with 25-m resolution in British Columbia, Canada, the method of mapping ecosites in this study would had an even lower costs and higher productivity in total/average value than of MacMillan *et al*.^14^. MacMillan *et al*.^14^ reported a low mapping costs ($0.2–3.5 ha^−1^) and a high productivity (0.15 to 2 × 10^6^ ha per person year), which were mostly spent in manually interpreting/digitizing satellite imagery and maps of ancillary environmental conditions but excluded the cost of the historical data that were used to create the heuristic rule base but did not be collected for the study. However, the cost and time for mapping ecosites would have grown several times more if map resolution changed from 25 to 10 m. In this study, mapping ecosites was done based on model-predicted SMR and SNR and the only costs were data processing associated with high-resolution DEM data and the majority of the time was used to calibrate/validate the models based on field data.

It was worth noting that a few field data were required when transferring the models to other large area, because field data were only used to re-calibrate/validate linear models but to re-calibrate/validate ANN models. The ANN models used in this study, including soil drainage models and soil texture model built to produce high-resolution soil property maps, were developed in a small watershed with 1450 ha (the BBW showed in Fig. 1) based on a huge number of data (more than 133,500 points coming from resampling a field-based soil map with 442 polygons)^41, 42, 50^. The linear models (simple linear regression equation) used in this study, including extended soil drainage models, extend clay content model, soil moisture regime model and soil nutrient regime model, were developed to adapt soil properties produced by the ANN models to fit field samples derived from large areas, and were calibrated/validated with field samples, 1507–1663 points^33, 44^. Furthermore, the minimum of required re-calibration/validation data for the linear models was far less than the field samples used. For example, extended soil drainage model included 12 simple linear regression equations that only required dozens of field data to re- calibrate/ validate the equations. It also was noticed that the models used in this study were parametrized based data from natural forest where stand conditions without artificial plantation or tree breeding. Thus, application of the results in plantations should be cautious and further improvements may be required to include the impacts of forest management activities. The method of mapping ecosites based on model-predicted SMR and SNR is easy to transfer to other large areas with a few field data, except managed forests.

## Conclusions

Model predictions of SMR and SNR were used to map forest ecosites for the entire province of Nova Scotia. Results indicated that by using SMR and SNR data alone, accuracy of ecosite classification was 61 and 59% for the Acadian- and Maritime-Boreal-region ecosites, respectively. Although inaccuracies in model predictions of SMR and SNR led to lowered replication of some ecosites the approach was easy to transfer to other large areas with a few field data, except managed forests. Results indicate that presented method has the potential to produce map forest ecosites with reasonable accuracy over large areas based on model predictions of SMR and SNR. Further studies to improve the accuracy of model predictions of SMR and SNR in areas of extreme conditions (e.g. Wet or Very Dry, Very Poor or Very Rich) could improve ecosite map accuracy.
